# A novel approach using nonlinear surfaces for dynamic aperture optimization in MBA synchrotron light sources

**DOI:** 10.1038/s41598-023-49979-1

**Published:** 2023-12-27

**Authors:** Edgar Andres Sanchez, Alain Flores, Jorge Hernandez-Cobos, Matías Moreno, Armando Antillón

**Affiliations:** 1 Instituto de Ciencias Físicas, Universidad Nacional Autónoma de México, Av. Universidad 1001, Col. Chamilpa, Cuernavaca, Morelos 62210 Mexico; 2 Departamento de Bioingeniería y Ciencias, Tecnológico de Monterrey, Puebla, 72453 Mexico; 3 Instituto de Física, Universidad Nacional Autónoma de México, Cd. de México, 04510 Mexico

**Keywords:** Mathematics and computing, Physics

## Abstract

MBA cell-based synchrotron light sources have enabled an unprecedented increase in beam coherence and brightness, greatly benefiting the scientific disciplines that rely on X-ray techniques. However, controlling the electron dynamics is a theoretical and technological challenge, due to the large number of parameters to adjust and constraints to satisfy when designing modern synchrotrons. Having versatile tools for the description and manipulation of electron dynamics could favor the design of these accelerators and lead to progress on several fronts in the understanding of matter. In this paper, a formalism based on the use of nonlinear geometric surfaces represented by polynomial quasi-invariants, to analyze and optimize the dynamic aperture of electrons in MBA storage rings, is introduced. The formalism considers on- and off-momentum particle dynamics. Within the optimization scheme, different objective functions defined in terms of the nonlinear surfaces, which are minimized using genetic algorithm methods, are proposed. A remarkable horizontal dynamic aperture exceeding 19 mm is obtained for the design particle of a synchrotron model with 86 pm $\cdot$ rad emittance along with a dynamic aperture above 5 mm for momentum deviations of ± 3$\%$. According to the results presented, this formalism could be greatly useful for manipulating the dynamical properties of electrons in synchrotrons light sources close to the diffraction limit.

## Introduction

The first direct observation of synchrotron radiation in the 1940s, carried out at the second synchrotron that became operational in the world^1^, triggered the observation of new structures in the absorption spectra of various elements^2^ and led to the widespread use of this radiation in the study of matter^3^. Although for several decades there were no accelerators fully dedicated to the production of synchrotron radiation, synchrotrons that were not only fully dedicated to the production of synchrotron light but were also optimized to increase the brightness of the beam came into operation in the 1990s^2^. These synchrotrons consist of arrays, called cells, of two or three magnetic dipoles separated by magnetic quadrupoles and free spaces (DBA and TBA cells, respectively), based on the proposal of Chasman and Green^4^. Many light sources using these schemes are still in operation in several countries^2^.

The demand for synchrotron light with higher brightness and coherence has driven the development of new models^5–9^. More specifically, the incorporation of MBA cells^10^, including multiple magnetic dipoles (more than 3) with different deflection angles, magnets with combined functions and intense quadrupoles, has allowed the development of synchrotron light sources at the diffraction limit^11^. This new type of accelerator reduces the horizontal emittance of the electron beam to below 100 pm   rad, which considerably increases the coherence and brightness of the light^11^. The development of new advanced technologies has made it possible to generate sophisticated MBA cells, allowing this new generation of synchrotrons to replace the previous ones. The commissioning of MAX-IV^12^, Sirius^13^, and ESRB-EBS^14^ are evidence of this progress.

However, as these new models actively seek to reduce natural emittance^15^, a plethora of magnets with different properties and parameters have been incorporated. Their effect on the electron beam goes beyond emittance reduction, making their optimal selection a major challenge. These elements, necessary in these designs, have the potential to strongly degrade the dynamic aperture and restrict the electron insertion process in the main ring; particularly, the use of intense quadrupoles increases the natural chromaticities of the synchrotron^16^. Intense magnetic sextupoles must also be incorporated to bring the chromaticities to zero, with the adverse effect of introducing nonlinearities in the electron dynamics. To reduce these effects, additional sextupoles and higher order magnetic multipoles are incorporated. The combination of multiple constraints and a high number of parameters to be adjusted results in higher demands on the design of the cell’s magnetic structure and, of course, technological challenges at various levels.

There are common methods for the optimization of the dynamic aperture of these synchrotrons; for example, the minimization of the resonant terms^17–21^ or the direct obtaining of the dynamic aperture by particle tracking^22–26^. However, since the reduction of nonlinear effects in these systems is usually very demanding, several strategies have been proposed and developed, generally based on the aforementioned methods. For example, the combined use of particle tracking and the calculation of resonant terms in optimization objectives^27^; the proposal of new optimization algorithms or the improvement of existing schemes^28–36^; and the analysis and minimization of fluctuations of conserved quantities in these systems have been explored^28,37,38^. Given the complexity of the problem, these methods usually employ multi-objective functions in which a considerable amount of time is spent to compute their arguments.

In this context, the use of approximate invariant polynomial roots has recently been proposed for the optimization of the nonlinear dynamics of these systems^39,40^. In particular, reference^40^ details a dynamic aperture optimization formalism for the design particle, using an objective function dependent on the real roots of the quasi-invariant polynomial of degree 5, since, according to that work, these approximately represent the trajectories of the system. Furthermore, in the above-mentioned study, the dynamic aperture of a storage ring with emittance above 1 nm $\cdot$ rad was optimized showing the advantages of using this type of polynomial in these processes. Whether these polynomials are useful for optimizing electron dynamics in MBA cell-based synchrotrons has not been studied and remained as a pending task.

In this work we present a novel formalism, based on the use of geometric surfaces constructed by quasi-invariants of motion, to study and optimize the dynamic aperture of electrons in a storage ring based on an MBA cell, for both the design particle and considering momentum deviations. This is achieved by extending the studies presented in references^39–43^ to propose an approximate polynomial invariant of degree 8 that is used in the construction of nonlinear surfaces, whose contour lines approximately represent the trajectories of the system for a given amplitude and momentum deviation. The concept of nonlinear surface is a fundamental element of the formalism, showing that the construction of quasi-invariants of motion in these systems is a versatile and powerful tool for the analysis and manipulation of their dynamical properties. This versatility is appreciated when, with relative easiness, nonlinear surfaces can be used to define several possible single-objective functions to optimize nonlinear dynamics, allowing a wide search of solutions in the parameter space, which is an advantage over other methods. Within the introduced optimization scheme, these surfaces are incorporated into the proposed single-objective functions that are optimized using genetic algorithm methods. The use of single-objective functions simplifies the optimization process, and it can be easily done with other numerical optimization methods, which is a significant advantage over other conventional nonlinear optimization schemes^44^.

For the cell design considered, in a configuration with an emittance of 86 pm $\cdot$ rad, a horizontal dynamic aperture greater than 19 mm is obtained for the design particle and greater than 5 mm for momentum deviations of $-\,3\%$ and $3\%$. The optimization was performed using a Ryzen-AMD desktop computer in a few hours of computational time. The reduced computer resources needed to perform these calculations is also an advantage over other methods.

These results have been corroborated using the OPA particle tracking module^45^. The optimal nonlinear multipoles were obtained only from the horizontal dynamics optimization. However, when these nonlinear multipoles are introduced in OPA, they generate larger dynamic apertures than when they are optimized with OPA. Based on the results obtained, this formalism could be extremely useful in the manipulation of the dynamical properties of electrons in synchrotrons in the diffraction limit and, therefore, push the frontier of synchrotron development.

## Revisiting the Courant-Snyder invariant and approximate constants of motion

In a synchrotron there are several periodic functions associated with the intensities of the different magnetic multipoles, such as the radius of curvature associated with the magnetic dipoles, and the quadrupole, sextupole, and octupole magnetic fields. They are usually represented by $\rho (s)$, *K*(*s*), *S*(*s*) and *O*(*s*), and satisfy the periodic conditions

1 $$\begin{aligned} \rho (s+c)= & {} \rho (s),\nonumber \\ K(s+c)= & {} K(s),\nonumber \\ S(s+c)= & {} S(s),\nonumber \\ O(s+c)= & {} O(s), \end{aligned}$$

where *c* indicates the period, which can be the circumference of the ring, the length of a cell, or a smaller distance inside the cell. As usual^16^, the functions (1) are written in terms of the functions $b_1(s)$, $b_2(s)$, $b_3(s)$ and $b_4(s)$, defined through the expression

2 $$\begin{aligned} b_m(s) = \frac{1}{B\rho }\frac{1}{(m-1)!}\frac{\partial ^{m-1}B_y(x,y)}{\partial x^{m-1}}\Big {|}_{y=0}, \end{aligned}$$

with $m=1,2,\ldots$, and $B\rho$ is the magnetic rigidity, which establishes the well-known relationship between the magnetic field, the radius of curvature and the electron energy $B\rho \, [\textrm{T}{\cdot }\textrm{m}] = 3.3356\, E[\textrm{GeV}]$. Furthermore, for a particle, the deviation of the magnitude of the momentum *p* with respect to the magnitude of the design momentum $p_0$ is written as $\Delta p \equiv p-p_0$ and it is proportional to the magnitude of the design moment, $\Delta p = \delta \; p_0$, where $\delta$ is the percentage of moment deviation with respect to the design moment, i.e. a dimensionless parameter.

### Nonlinear surfaces with chromatic effects

Reformulating the discussion found in papers^39–43^ with a geometric point of view, the Courant-Snyder invariant

3 $$\begin{aligned} I_0 = \gamma _x(s)\, x^2 + 2 \alpha _x(s)\, x\, p_x + \beta _x(s)\, p_x^2, \end{aligned}$$

is a conserved quantity in the one-dimensional linear system described by the Hamiltonian

4 $$\begin{aligned} H_0 = \frac{1}{2} \left( p_x^2 + K(s)\, x^2\right) , \end{aligned}$$

which allows the definition of the surface

5 $$\begin{aligned} S_{0}(x,p_x)=\gamma _x(s)\, x^2 + 2 \alpha _x(s)\, x\, p_x + \beta _x(s)\, p_x^2, \end{aligned}$$

which we will call Courant-Snyder surface and describes an elliptic paraboloid whose principal axes depend parametrically on *s*. Once these are determined for a fixed point $s_0$ in the ring, the initial conditions $(x_0,p_{x_0})$ define a particular level curve, associated to a fixed value of the function (5): $S_{0}(x_0,p_{x_0})=I_0=\gamma _x(s_0)\, x^2 + 2 \alpha _x(s_0)\, x\, p_x + \beta _x(s_0)\, p_x^2$ (which is equivalent to a fixed amplitude of movement). It is well known that the dependence of the principal axes of this elliptic paraboloid on the *s* parameter (position in the ring) is limited by the periodic conditions imposed on the coefficients appearing in the quadratic function (5) (Courant-Snyder parameters)

6 $$\begin{aligned} \alpha _x(s+c)= & {} \alpha _x(s), \nonumber \\ \beta _x(s+c)= & {} \beta _x(s), \nonumber \\ \gamma _x(s+c)= & {} \gamma _x(s). \end{aligned}$$

From this perspective, although the principal axes of the elliptic paraboloid oscillate, the dynamics of the system (4) guarantees that the contour line defined by the equation $S_{0}(x,p_x)=I_0$, for a given value of $I_0$, encloses a constant area as *s* changes along the trajectory of the ring, despite changing its shape.

In the same way, following the description given in works^39,40,42,43^, the two-dimensional, nonlinear system with chromatic effects, described by the Hamiltonian^17^

7 $$\begin{aligned} H(x,p_x,y,p_y,s){} & {} = \frac{1}{2}(p_x^2 + p_y^2)\left( 1 - \delta + \delta ^2 + \cdots \right) \nonumber \\{} & {} \quad -\,\, b_1(s) \, x\, \delta + \frac{b_1^2(s)}{2} x^2 + \frac{b_2(s)}{2} \left( x^2 - y^2\right) \nonumber \\{} & {} \quad + \,\,\frac{b_3(s)}{3} \left( x^3 - 3xy^2\right) + \frac{b_4(s)}{4}\left( x^4-6x^2y^2+y^4\right) \nonumber \\{} & {} \quad +\,\, \cdots , \end{aligned}$$

has the approximate invariant

8 $$\begin{aligned} I=\sum _{n=0} \sum _{\begin{array}{c} i+j+k+l\ge 2\\ i,j,k,l=0 \end{array}} A^{(n)}_{ijkl}(s) x^ip_x^jy^kp_y^l\delta ^n, \end{aligned}$$

where the functions $A^{(n)}_{ijkl}(s)$, that generalize the coefficients of the polynomial (3), i.e., $\alpha _x$, $\beta _x$, $\gamma _x$, are also subject to periodic conditions

9 $$\begin{aligned} A^{(n)}_{ijkl}(s+c) = A^{(n)}_{ijkl}(s). \end{aligned}$$

Similar to the Courant-Snyder case, we can geometrically interpret the approximate invariant (8) as the hypersurfaces

10 $$\begin{aligned} f(x,p_x,y,p_y,\delta ){} & {} \nonumber \\ =\sum _{n=0}{} & {} \sum _{\begin{array}{c} i+j+k+l\ge 2\\ i,j,k,l=0 \end{array} } A^{(n)}_{ijkl}(s) x^ip_x^jy^kp_y^l\delta ^n, \end{aligned}$$

which are parametrically dependent on $\delta$. It is worth noting that, for a fixed value of $\delta$, the surface resulting from the projection of the hypersurface (10) onto the horizontal phase space ($x,p_x$) for a fixed value of *I* has associated contour lines that will not necessarily be simple closed curves. The nonlinearity of the system, introduced by means of the $b_m(s)$ coefficients with $m>2$, implies that there will be non-zero $A^{(n)}_{ijkl}$ coefficients with $i+j+k+l>2$, which, as is well known, will result in the aforementioned hypersurface (10) projections (at fixed $\delta$) having cubic and higher order contour lines, which in general represent unbounded motion. However, studies^39,40^ have shown that in the presence of nonlinearities, it is possible to obtain closed contour lines for hypersurface (10) projections (fixed $\delta$) in horizontal phase space, under certain conditions and for some values of *I*; i.e., for certain oscillation amplitudes. These curves represent bounded motions of the system and, analogous to the Courant-Snyder case, although the coefficients $A^{(n)}_{ijkl}$ oscillate along the ring, the dynamics of the system (7) allows the area enclosed by each contour line (defined by a fixed value of *I*) to be approximately conserved.

Due to the context of the problem, we will refer to the projections of the hypersurface (10) onto one of the phase spaces and a fixed $\delta$ value, as nonlinear surfaces $S^\delta (x,p_x)$. The remainder of this paper will only deal with nonlinear surfaces in horizontal phase space. The fact that the oscillation amplitudes in the horizontal plane are larger than in the vertical one suggests that the dynamics of the system will be more complex in the horizontal than in the vertical plane and, from a physical point of view, it is worth focusing on the horizontal dynamics to advance the understanding of the problem.

### Quasi-invariant for a nonlinear, chromatic, two-dimensional system

Following the formalism proposed in works^39–43^, the equations of motion satisfied by the functions $A^{(n)}_{ijkl}(s)$ are obtained by imposing the invariance condition

11 $$\begin{aligned} \frac{dI}{ds} = \{I,H\}+ \frac{\partial {I}}{\partial {s}}=0, \end{aligned}$$

to the expression (8), under the dynamics of the system (7). In this way it is possible to obtain a system of linear differential equations (LDE) whose number of participating functions depends on the degree of the polynomial considered. In order to be able to describe accurately complex structures of resonances in phase space a polynomial of degree 8 is considered in this work, instead of the degree 5 polynomial used in ref.^40^.

A system of 418 equations was obtained using wxMaxima^46^ to manipulate this polynomial of degree 8 and at first order in $\delta$. The complete system of equations is not included in this manuscript for ease of reading, but can be consulted in the supplementary material (SM). It should be noted that in this LDE system the equations

12.1 $$\begin{aligned} \frac{d A^{(0)}_{2000}}{ds}&= \left( b_2 + b_1^{2}\right) A^{(0)}_{1100}, \end{aligned}$$

12.2 $$\begin{aligned} \frac{d A^{(0)}_{1100}}{ds}&= - 2 A^{(0)}_{2000} + 2 \left( b_2 + b_1^{2}\right) A^{(0)}_{0200}, \end{aligned}$$

12.3 $$\begin{aligned} \frac{d A^{(0)}_{0200}}{ds}&= - A^{(0)}_{1100}, \end{aligned}$$

appear, reproducing the $\alpha _x$, $\beta _x$ and $\gamma _x$ equations, which are important for the consistency of this formalism with the linear case. In addition, the lowest order nonlinear coefficients $A^{(0)}_{ijkl}$, satisfy the equations

13.1 $$\begin{aligned} \frac{d A^{(0)}_{1200}}{ds}&= 3( b_{1}^{2} + b_{2} )A^{(0)}_{0300 } - 2 A^{(0)}_{2100 }, \end{aligned}$$

13.2 $$\begin{aligned} \frac{d A^{(0)}_{2100}}{ds}&= 2( b_{1}^{2} + b_{2}) A^{(0)}_{1200} + 2 b_{3} A^{(0)}_{0200} - 3 A^{(0)}_{3000} , \end{aligned}$$

14.1 $$\begin{aligned} \frac{d A^{(0)}_{3000}}{ds}&= (b_{1}^{2} + b_{2} )A^{(0)}_{2100 } + b_{3} A^{(0)}_{1100 } , \end{aligned}$$

14.2 $$\begin{aligned} \frac{d A^{(0)}_{0300}}{ds}&= - A^{(0)}_{1200}, \end{aligned}$$

15.1 $$\begin{aligned} \frac{d A^{(0)}_{0111}}{ds}&= -2 b_{2} A^{(0)}_{0102} - 2 A^{(0)}_{0120} - A^{(0)}_{1011}, \end{aligned}$$

15.2 $$\begin{aligned} \frac{d A^{(0)}_{1011}}{ds}&= ( b_{1}^{2} + b_{2} ) A^{(0)}_{0111} - 2 b_{2} A^{(0)}_{1002 } - 2 A^{(0)}_{1020}, \end{aligned}$$

15.3 $$\begin{aligned} \frac{d A^{(0)}_{1020}}{ds}&= (b_{1}^{2} + b_{2}) A^{(0)}_{0120 } - b_{2} A^{(0)}_{1011 } -b_{3} A^{(0)}_{1100 } , \end{aligned}$$

15.4 $$\begin{aligned} \frac{d A^{(0)}_{1002}}{ds}&= (b_{1}^{2} + b_{2} )A^{(0)}_{0102 } - A^{(0)}_{1011 }, \end{aligned}$$

15.5 $$\begin{aligned} \frac{d A^{(0)}_{0120}}{ds}&= -b_{2} A^{(0)}_{0111 } - 2 b_{3} A^{(0)}_{0200 } - A^{(0)}_{1020 } , \end{aligned}$$

15.6 $$\begin{aligned} \frac{d A^{(0)}_{0102}}{ds}&= -A^{(0)}_{0111 } - A^{(0)}_{1002 }, \end{aligned}$$

Only coefficients related to the horizontal dynamics of the accelerator appear in Eqs. (13–14), while in Eq. (15) couplings between coefficients associated with both horizontal and vertical space are observed. Within the LDE system, there are many such couplings. On the other hand, the lowest order coefficients of chromatic character $A^{(1)}_{ijkl}$ satisfy the expressions

16.1 $$\begin{aligned} \frac{d A^{(1)}_{1000 }}{d s}&= (b_{1}^{2} + b_{2}) A^{(1)}_{0100 } - b_{1} A^{(0)}_{1100 } , \end{aligned}$$

16.2 $$\begin{aligned} \frac{d A^{(1)}_{0100 }}{d s}&=-\, 2 b_{1} A^{(0)}_{0200 } - A^{(1)}_{1000 },\end{aligned}$$

16.3 $$\begin{aligned} \frac{d A^{(1)}_{1100 }}{d s}&= 2 ( b_{1}^{2} + b_{2}) A^{(1)}_{0200 } - 2 b_{1} A^{(0)}_{1200 } + 2 A^{(0)}_{2000 } \nonumber \\&-\,2 A^{(1)}_{2000 } ,\end{aligned}$$

16.4 $$\begin{aligned} \frac{d A^{(1)}_{0200 }}{d s}&=-\, 3 b_{1} A^{(0)}_{0300 } + A^{(0)}_{1100 } - A^{(1)}_{1100 } ,\end{aligned}$$

16.5 $$\begin{aligned} \frac{d A^{(1)}_{2000 }}{d s}&= (b_{1}^{2} + b_{2} )A^{(1)}_{1100 }- b_{1} A^{(0)}_{2100 } + b_{3} A^{(1)}_{0100 } , \end{aligned}$$

16.6 $$\begin{aligned} \frac{d A^{(1)}_{0020 }}{d s}&=-\,b_{1} A^{(0)}_{0120 } - b_{2} A^{(1)}_{0011 } - b_{3} A^{(1)}_{0100 } ,\end{aligned}$$

16.7 $$\begin{aligned} \frac{d A^{(1)}_{0011 }}{d s}&=-\, b_{1} A^{(0)}_{0111 } - 2 b_{2} A^{(1)}_{0002 } - 2 A^{(1)}_{0020 } ,\end{aligned}$$

16.8 $$\begin{aligned} \frac{d A^{(1)}_{0002 } }{d s}&= - \,b_{1} A^{(0)}_{0102 } - A^{(1)}_{0011 } , \end{aligned}$$

i.e., the coefficients incorporating corrections for the description of off-momentum particles. Note that although these equations contain nonlinear parameters ($$b_3$$), in the linear limit ($$b_n\rightarrow 0$$ for $$ n>2$$) they admit nontrivial solutions.

It is important to consider that these functions will take real and bounded values only if an appropriate selection of $b_n$ functions is made. In general, an arbitrary selection of $b_n$ functions could produce unstable solutions for the $A^{(n)}_{ijkl}$ functions. Due to the complexity of the 418-dimensional system, finding an analytical solution will require a considerable amount of computational time; therefore, it has been numerically solved in this work imposing the periodic conditions (9).

It is worth outlining the procedure to determine the functions $A^{(0)}(s)$ and the chromatic functions $A^{(1) }(s)$ used in this work. Let us consider a column vector *W*(*s*) with 249 components $A^{(0)}_{ijkl}(s)$ followed by 169 components $A^{(1)}_{ijkl}(s)$, which are determined by the 418 differential equations depicted in **SM**. A small set of these equations is shown in Eqs. (12–16).

These differential equations can be written in the form

17 $$\begin{aligned} \dot{W}(s) = M\ W(s) \end{aligned}$$

where *M* is a square matrix that depends on dipoles, quadrupoles, sextupoles, and other magnetic multipoles strengths, and can be obtained for each magnetic element *k*, where the magnetic strengths are considered to be constant. In this case, the general solution of Eq. (17), for constant $M_k$, is

18 $$\begin{aligned}W(s_2) &= T_k(s_2,s_1)\ W(s_1),\\ {\rm with}\ \ T_k(s_2,s_1)&= \exp (M_k(s_2-s_1)). \end{aligned}$$

Matrices $T_k$, corresponding to the various elements present in the synchrotron cell, can be multiplied in the appropriate order to obtain the transport matrix for the entire cell of *p* elements, $T_c = T_pT_{p-1} \cdots T_2T_1$. Considering the periodic boundary conditions $A^{(0)}_{ijkl}(s)=A^{(0)}_{ijkl}(s+c)$ and $A^{(1)}_{ijkl}(s)=A^{(1)}_{ijkl}(s+c)$, it can be written

19 $$\begin{aligned} W(c) = T_c\ W(0)=W(0), \end{aligned}$$

representing an eigenvalue problem that can be solved for *W*(0) if

20 $$\begin{aligned} \det [T(c)-I]=0. \end{aligned}$$

Then, Eq. (19) allows to find the values of the periodic functions $A^{(0)}_{ijkl}(s=0)$ and $A^{(1)}_{ijkl}(s=0)$ contained in the vector *W*, at $s=0$.

## Geometric approximation in obtaining bounded motion in phase space

In Ref.^40^ it has been shown that it is possible to increase the stable area in the horizontal phase space of electrons in a synchrotron using polynomial quasi-invariants of motion. The proper classification of the real roots obtained from the quasi-invariant polynomial of order 5 into branches poses a serious problem in that work. When this was possible with small oscillation amplitudes, a sextupole optimization could be performed by forcing the chosen branches of the quasi-invariant real roots to topologically approximate the ellipse described by the linear motion through the Courant-Snyder invariant. The procedure for obtaining stable trajectories and resonances is described in Ref.^39^.

**Figure 1:**
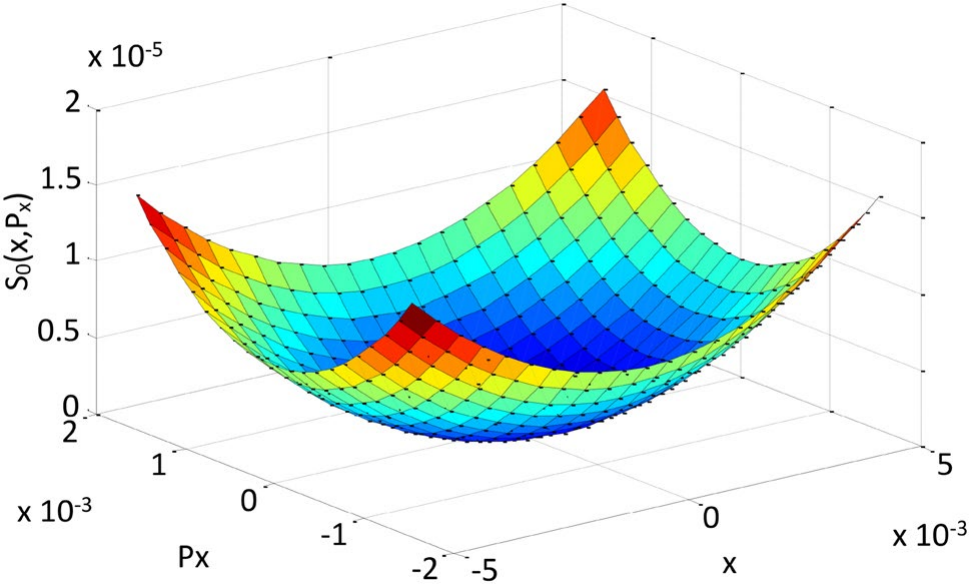
Linear surface corresponding to the Courant-Snyder invariant at the start of the unit cell. Colors correspond to different values of the invariant $S_0(x,p_x)$. Axes units are [m] and [rad], corresponding to the coordinate *x* and conjugate momentum $p_x$ of the horizontal phase space, and [m rad] for $S_0(x,p_x)$.

In this paper we present a different approach to the above process, i.e., inducing a similarity between the topology of nonlinear and linear phase spaces. To solve the classification of roots difficulty we have opted for the introduction of geometric surfaces described by the quasi-invariant, which seems to be a promising idea according to the results shown below. For this purpose, we will consider the Courant-Snyder (linear) surface $S_0$ represented by Eq. (5). By constructing a grid over the horizontal phase space and calculating the value of the invariant as a function of *x* and $p_x$ on the grid, we obtain this surface as shown in Fig. 1.

**Figure 2:**
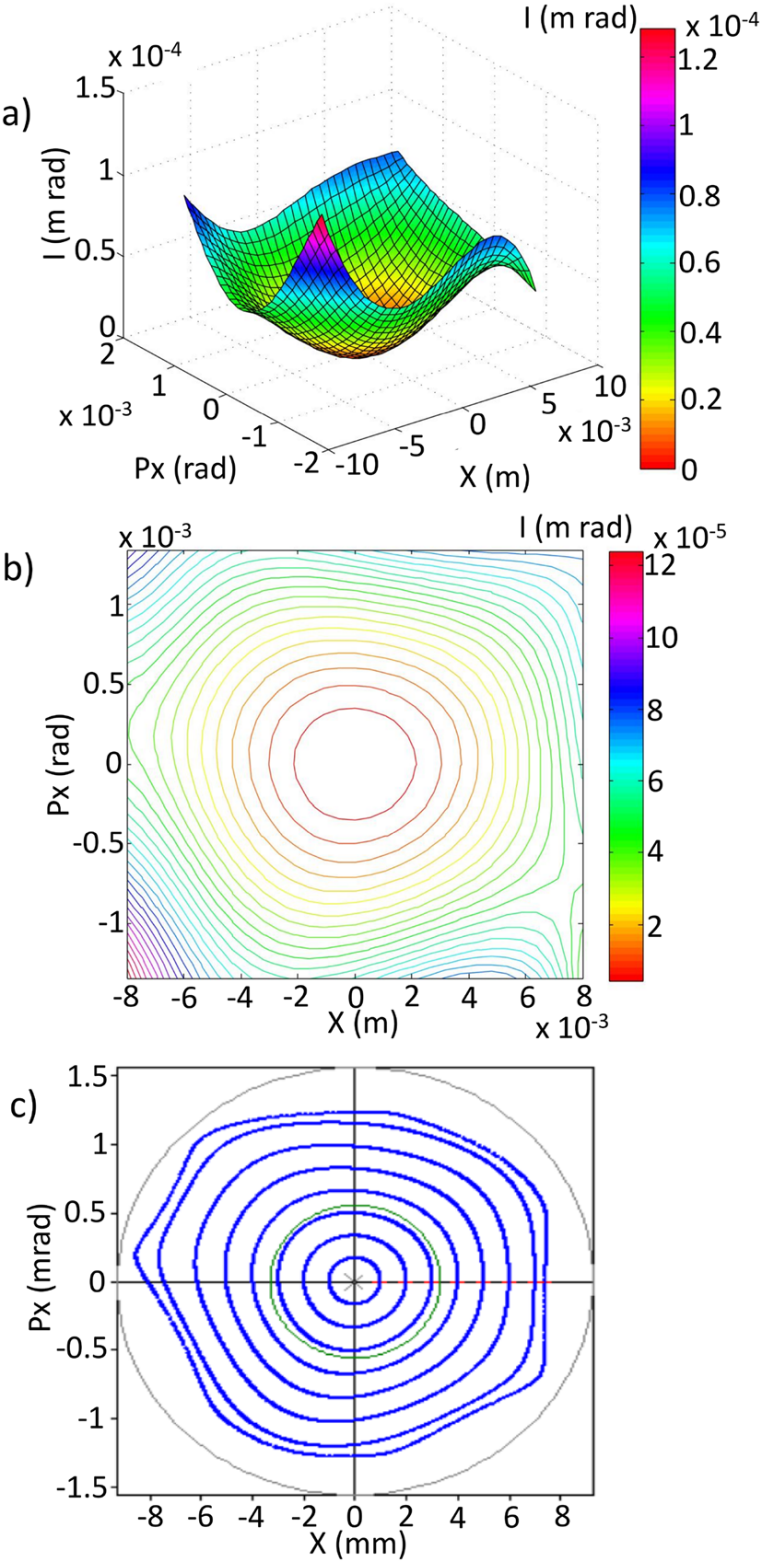
(**a**) Representative nonlinear surface constructed from a polynomial quasi-invariant of degree 8 corresponding to a phase space for a momentum deviation $\delta$. (**b**) Contour lines of the previous surface. (**c**) Phase space of the same system obtained with the OPA particle tracking module.

The shape of the nonlinear surface, which results from the invariant of Eq. (10), depends on the values of the functions $A^{(n)}_{ijkl}$ that contribute the most to the expression of the invariant, and are very sensitive to the configuration of the nonlinear magnetic elements of the cell. Figure 2 is an example of this type of surface, restricted to the horizontal phase space, i.e., $y=p_y=0$.

### Contour lines of nonlinear surfaces

Once the coefficients $A^{(n)}_{ijkl}$ of the polynomial function (10) have been determined for a given value of *s*, these coefficients can be used to represent nonlinear surfaces for a fixed value of $\delta$. The procedure to achieve this representation will depend on the computational resources available. In this work we have used the *surf* function of the MATLAB^®^ numerical computation environment to generate the 3-D surface data and the *contour* function of the same environment to generate the 2-D contour lines. The contour lines of one of these surfaces, for a fixed value of *I*, approximately represent the horizontal phase space of the nonlinear chromatic system (7) to order $\delta$ in this work.

As already mentioned, in general, contour lines of nonlinear surfaces do not represent bounded motions in the horizontal phase space, however, in this and the following section protocols to promote the existence of closed contour lines on nonlinear surfaces for different values of $\delta$, under certain conditions, will be described. These protocols are based on optimization schemes that employ the Courant-Snyder surface $S_{0}(x,p_x)$ to impose constrictions on the nonlinear surface $S^{\delta }(x,p_x)$, without resorting to the identification of real root branches described in reference^40^, thus avoiding such difficulties.

In this and the next section we consider a grid of dimension $n \times n$, defined over the horizontal phase space, at whose vertices the values of the Courant-Snyder and nonlinear surfaces are computed. Optimizations are studied for moment deviation percentages $\delta$ in the range $-\delta _m\le \delta \le \delta _m$, with $\delta _m>0$ the maximum percentage considered.

### The proposal of a geometric objective function

In this section two objective function schemes of geometric nature are explored, more robust and simpler to use than the one proposed in Ref.^40^.

**Using separation between surfaces**

A simple way to define an objective function is to quantify the separation between the surfaces in Figs. 1 and 2 in each grid point and then sum up all these values. This is done for each of the *N* phase spaces corresponding to the several momenta values $\delta$ considered.

This can be done by means of the following expression

21 $$\begin{aligned} f^\delta _{obj1}= \sum _{j=1}^{n}\sum _{i=1}^{n}{\big |S^\delta (x_i,p_{xj})-S_{0}(x_i,p_{xj})\big |}, \end{aligned}$$

where $S^\delta (x_i,p_{xj})$ and $S_{0}(x_i,p_{xj})$ represents the non linear and linear surfaces evaluated at points $(x_i,p_{xj})$ of the grid.

For the $\delta =0$ case the linear surfaces correspond to the Courant-Snyder surface $S_0(x,p_x)$, given by Eq. (5). To compare a nonlinear surface for $\delta \ne 0$ with the linear surface for $\delta = 0$, all the *x* values of the grid $(x,p_x)$ are displaced to $x+D(s)\delta$, where *D*(*s*) is the dispersion function. The nonlinear surface is evaluated using this displaced grid, and then centered at the origin, where the comparison is made.

The definition of $f^\delta _{obj1}$ in Eq. (21) includes the case for the on-momentum reference particle, i.e, at $\delta =0$, as well as terms corresponding to off-momentum particles ($\delta \ne 0$). These cases will be analyzed separately below.

In minimizing $f^\delta _{obj1}$, we seek for solutions of the nonlinear problem that are as close as possible to the linear surface. As mentioned before, the nonlinear surface is intended to resemble that of the linear case for small amplitudes.

The objective function $f^\delta _{obj1}$ defined in the expression (21) considers the sum of distances between the linear and nonlinear surfaces, for each grid point, for a fixed $\delta$ phase space. Since these distances depend on the nonlinear functions $A^{(0)}_{ijkl}(s)$ and $A^{(1)}_{ijkl}(s)$, the terms that deform the phase space the most will be penalized the most. Being the only function that is intended to be optimized, the inclusion of weights in the problem is not necessary. We consider this to be a strength of the algorithm by using the objective function of Eq. (21).

**Using Gauss curvature**

A geometrical parameter that can be used as an alternative in the process of optimizing the dynamic aperture in the synchrotron light source is the Gaussian curvature $K_G$. First, it is necessary to know this for a linear surface such as the one shown in Fig. 1. For this purpose, it can be observed that the elliptic paraboloid with semi-axis *a*, *b* given by the quadratic form^47^

22 $$\begin{aligned} z{(v,w)}=(v/a)^2+(w/b)^2, \end{aligned}$$

can be approximately expressed as

23 $$\begin{aligned} z{(v,w)}=\frac{1}{2} \left( \kappa _1v^2+\kappa _2w^2 \right) \end{aligned}$$

in a neighborhood of the origin (large enough for an MBA lattice optimization), where $\kappa _1$ and $\kappa _2$ are the principal curvatures. The Gaussian curvature $K_G$ is connected with them through

24 $$\begin{aligned} K_G=\kappa _1\kappa _2. \end{aligned}$$

Considering the approximation of Eq. (23) and that, in a symmetry point of the magnets cell,

25 $$\begin{aligned} I = \gamma _x(0)\, x^2 + \beta _x(0)\, p_x^2, \end{aligned}$$

comparing the expressions (23), (25) and (24), it follows that

26 $$\begin{aligned} \kappa _1= 2\gamma _x(0),\,\, \kappa _2=2\beta _x(0),\,\, K_G=4. \end{aligned}$$

This last parameter can be a useful guide in the search for a good dynamic aperture in a light source. It can also be useful as a secondary tool after using the objective function $f_{obj1}^\delta$. Linked to this geometrical parameter, a second objective function is defined as

27 $$\begin{aligned} f_{obj2}^\delta = \sum _{j=1}^{n} \sum _{i=1}^{n}\mid K^{\delta }_G(x_i,p_{xj})-4\mid , \end{aligned}$$

where $K^{\delta }_G(x_i,p_{xj})$ represents the Gauss curvature surface of the nonlinear surface (Eq. 10), evaluated at points $(x_i,p_{xj})$ of the considered grid. The curvature $K_{Gauss}$ has been evaluated using the Matlab function *surfature*^48^ through expression

28 $$\begin{aligned} K_{Gauss} = \frac{LN - M^2}{EG - F^2}, \end{aligned}$$

where *E*, *F*, *G* are coefficients of the first fundamental form, and *L*, *M*, *N* are coefficients of the second fundamental form. If $U \subset {\mathbb {R}}^2$, and

29 $$\begin{aligned}&h: U \rightarrow {\mathbb {R}} \nonumber \\&\quad (x,p_x)\mapsto h(x,p_x), \end{aligned}$$

where *h* represents a nonlinear surface $S^\delta$, then, the coefficients are given by

30 $$\begin{aligned} E{} & {} =1+{h^{2}_{x}},\;\; F=h_{x}{h_{p_{x}}}, \;\; G=1+h_{p^{2}_{x}}, \nonumber \\ L{} & {} =\frac{h_{xx}}{\sqrt{1+{h^{2}_{x}} +h_{p^{2}_{x}}}}, \; \; M=\frac{h_{x}{h_{p_{x}}}}{\sqrt{1+{h^{2}_{x}} +{h_{p^{2}_{x}}}}}, \nonumber \\ N{} & {} =\frac{h_{{p_{x}p_{x}}}}{\sqrt{1+{h^{2}_{x}} +h_{{p}^{2}_{x}}}}, \end{aligned}$$

where a subindex letter means derivative with respect to that variable^49^.

At another point of the accelerator $s=s_1$, the Courant-Snyder invariant can have the general form

31 $$\begin{aligned} I=\gamma _x(s_1) x^2+2\alpha _x(s_1) xp_x+\beta _x(s_1)p_x^2. \end{aligned}$$

Through the transformation

32 $$\begin{aligned} \begin{pmatrix} x \\ p_x \end{pmatrix} =\begin{pmatrix} \cos \theta &{} -\beta _0\sin \theta \\ \frac{1}{\beta _0}\sin \theta &{}\cos \theta \end{pmatrix}\begin{pmatrix} x^\prime \\ p_x^\prime \end{pmatrix} \end{aligned}$$

where $\beta _0=\beta _x(0)$, the Eq. (31) can be written in the form

33 $$\begin{aligned} I=A x^{'2}+B p_x^{'2}, \end{aligned}$$

where A and B are functions of $\alpha$, $\beta$ and $\gamma$, provided that $\theta$ is chosen in the form

34 $$\begin{aligned} \tan 2\theta =\frac{2\alpha (s_1)}{\beta _0\gamma (s_1)-\gamma _0{\beta (s_1)}} \end{aligned}$$

to cancel the term $x^{'}p_x^{'}$. From the expression (34) it also follows that

35 $$\begin{aligned} A= \frac{1}{2}\kappa ^{'}_1, \,\, B= \frac{1}{2}\kappa ^{'}_2. \end{aligned}$$

Using (34) and (35) it can be shown that

36 $$\begin{aligned} K^{'}_G=\kappa ^{'}_1\kappa ^{'}_2=4, \end{aligned}$$

and thus

37 $$\begin{aligned} K^{'}_G=K_G, \,\,\,\forall s\,\,\, \text {in the ring}, \end{aligned}$$

showing that the Gauss curvature of the Courant-Snyder surface is constant, at this level of approximation, at any point *s* of the synchrotron ring.

In this paper we will refer to the surfaces $S^\delta (x_{i},p_{xj})$ associated to the quasi-invariant as nonlinear surfaces and in the case of the corresponding Gaussian curvature surfaces $K^{\delta }_G(x_i,p_{xj})$, as associated nonlinear surfaces and, in general, simply as surfaces, when there is no ambiguity.

## Application to an MBA synchrotron of the nonlinear-surfaces based objective functions

The use of the methodology described above to achieve good dynamic performance in MBA cell models employed in synchrotron sources in the diffraction limit is studied in this section.

### An MBA lattice for the Mexican synchrotron light source combining the ESRF and SLS models, a case of study

**Figure 3:**
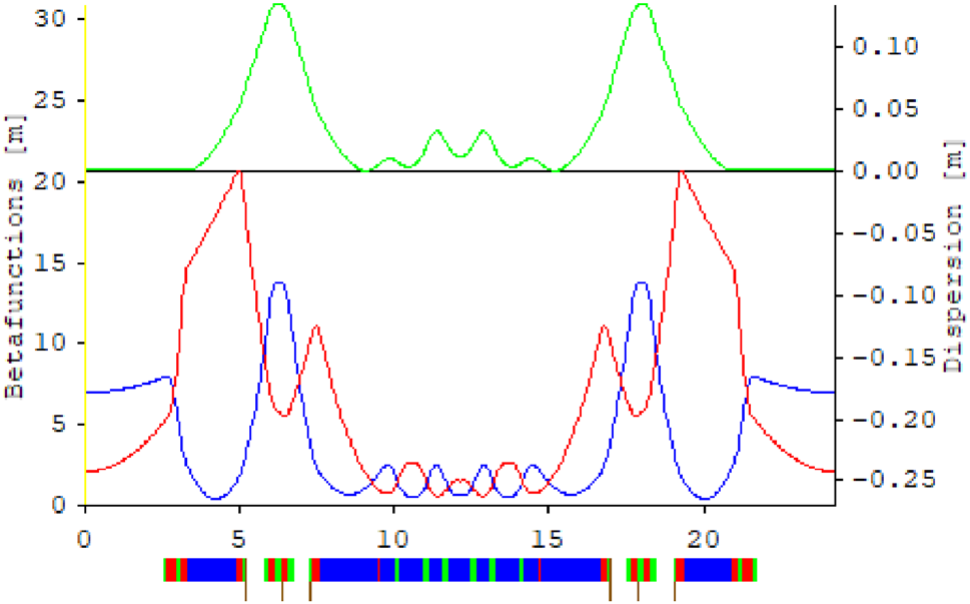
Optical functions of an MBA cell in the synchrotron ring considered. The functions $\beta _x(s)$ and $\beta _y(s)$ are respectively marked in blue and red and the dispersion function $\eta (s)$ is marked in green. The lower part shows the distribution of dipoles (blue), quadrupoles (red), sextupoles (green); and octupoles are represented with brown vertical lines of the MBA cell.

**Figure 4:**
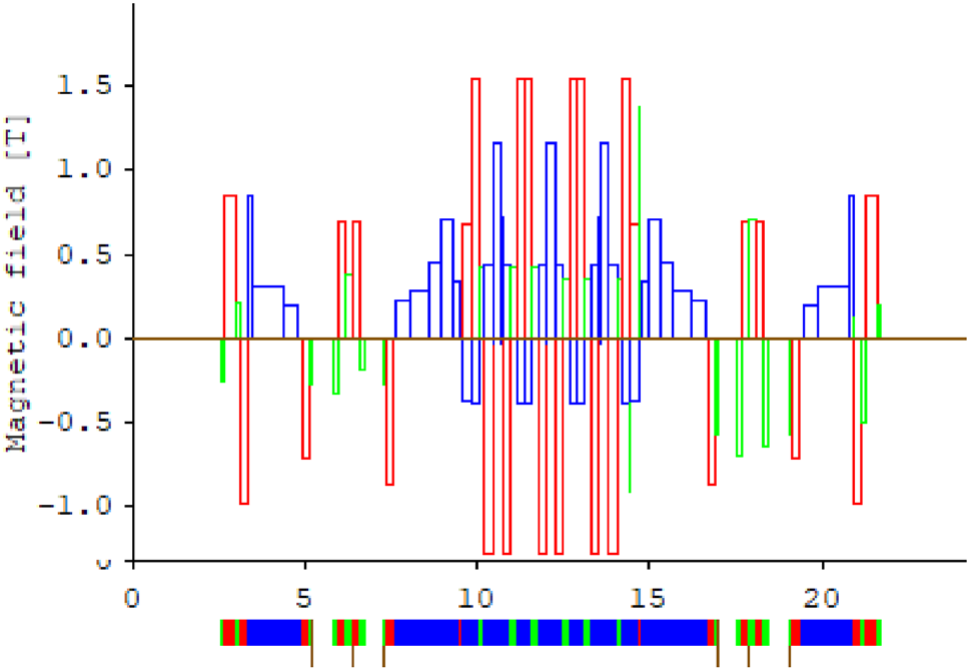
Magnetic fields of the magnets in Fig. 3 are shown. The left set of magnets, separated by drift spaces, shown in the bottom of the figure, corresponds to a matching cell. The field strengths for the corresponding dipoles (blue), quadrupoles (red), and sextupoles (green) are depicted in the upper part of the figure. These field values are related with the parameters $b_n$ through Eq. (2).

In search of an appropriate design for the Mexican light source, a combination of novel aspects, such as the use of LBG^14,50^, of the European ESRF-EBS synchrotron and the design contemplated for the upgrade of the Swiss synchrotron, SLS-2, has been considered. The section that includes a dispersion bump is taken from the European ESRF-EBS synchrotron, and the the internal cells that have been optimized with reverse bends are taken from the Swiss synchrotron, SLS-2. Both models, with emittances of the order of 130 pm$\,\cdot \,$rad, are considered fourth-generation storage rings^51^. A cell of the model under study is shown in Figs. 3 and 4.

The linear optical functions for one cell are shown in Fig. 3. The MBA cell is shown at the bottom of the figure, where dipoles, quadrupoles, and sextupoles are depicted in blue, red, and green, respectively. The complete ring has 20 cells. In Fig. 4 the cell is shown in more detail, depicting the magnetic fields of each magnet, following the magnets color code.

Once the dipole and quadrupole magnetic fields have been fixed, the storage ring optical functions and main parameters are determined; the latter are shown in Table 1. Natural chromaticities indicate that the sextupole intensities should not be very large, therefore, when performing chromaticity corrections, undesirable nonlinear phenomena should be minimal.

This synchrotron model based on an MBA cell represents a suitable problem to study with this methodology due to the complexity of its design, its low emittance and the high number of parameters to be optimized.

**Table 1:** Main parameters of the storage ring, which unit cell is shown in Figs. 3 and 4, considered as the starting point.

Parameter	Value
Energy	$3 \; \textrm{GeV}$
Circumference	$485.016 \; \textrm{m}$
cells	$20 \;$
Betatron tunes $(\nu _x, \nu _y)$	48.802, 27.653
Natural chromaticities $(\xi _x, \xi _y)$	$-\,86.70, -\,64.01$
Emittance $(\varepsilon _x)$	$113 \; \textrm{pm} \cdot \textrm{rad}$
Moment compaction factor $(\alpha _c)$	$1 \times 10^{- 4}$

### Using objective function $f_{obj1}^\delta$: optimizing parameters for the phase space corresponding to $\delta =0$

In this section we are going to study the MBA lattice, of the type of Figs. 3 and 4 at various operating points under the nonlinear optimization scheme using the objective function $f_{obj1}^\delta$ of Eq. (21).

In the general scheme of Fig. 3, from which this model and others shown later arise, 20 sextupoles (boldface) and 6 octupoles (underline) have been arranged in the list (38), among the other elements involved in the cell of Figs. 3 and 4. It has been deliberately decided not to use a default symmetry scheme of sextupole and octupole families, mainly to explore the potential of the method with a large number of optimization parameters, as well as to reduce most of the nonlinear effects produced by sextupoles in a single cell^52^.

38 $$\begin{aligned} \begin{aligned}D1, {\textbf {SE1}}, QF1, {\textbf {FD2}}, QD2, {\textbf {FD3}}, A5, A4, A3, A2, A1,\\ D4, QD3, {\textbf {SD1}}, \underline{O2}, D5D6, {\textbf {S1}}, QF4, {\textbf {SF1}}, \underline{O1}, QF4,\\ {\textbf {S2}}, D9D10, \underline{O3}, {\textbf {SD1}}, QD5, D11, B1, B2, B3, B4, B5, D12, \\ QF7, {\textbf {SD3}}, DQ6, {\textbf {S1S}}, ABQ1, {\textbf {S2S}}, DQ1S, B1S, B2S, B3S, \\ B2S, B1S, DQ1S, {\textbf {S2S}}, ABQ1, {\textbf {S1SH}}, {\textbf {S1SH}}, ABQ1, {\textbf {S2S}}, \\ DQ1S, B1S, B2S, B3S, B2S, B1S, DQ1S, {\textbf {S2S2}}, ABQ1, \\ {\textbf {S1SH2}}, {\textbf {S1SH2}}, ABQ1, {\textbf {S2S2}}, DQ1S, B1S, B2S, B3S, \\ B2S, B1S, DQ1S, {\textbf {S2S2}}, ABQ1, {\textbf {S1S2}}, DQ6, {\textbf {SD32}}, QF7, \\ D12, B5, B4, B3, B2, B1, D11, QD5, {\textbf {SD2}}, \\ \underline{O31}, D9D10, {\textbf {S4}}, QF4, \underline{O11}, {\textbf {SF2}}, QF4, {\textbf {S3}}, \\ D5D6, \underline{O21}, {\textbf {SD2}}, QD3, D4, A1, A2, A3, A4, A5, \\ {\textbf {FD32}}, QD2, {\textbf {FD22}}, QF1, {\textbf {SE2}}, D1. \end{aligned} \end{aligned}$$

These 26 parameters are adjusted during the optimization process, i.e., they are considered free parameters to optimize the phase space(s). The sextupoles **SF1** and **SD1** are adjusted in each optimization run to keep the chromaticities close to zero, $\xi _x$, $\xi _y \approx 0$. From Table 1, the ring emittance is 113 pm$\,\cdot \,$rad, the tunes are $\nu _x=48.802$ and $\nu _y=27.653$. The selection of quadrupoles produces a phase advance of $\Delta \varphi _x=2.9998\, \pi$ and of $\Delta \varphi _y=1.9984\, \pi$, between the dispersion bumps of the cell, close to a $-I$ transformation^53,54^ for the horizontal direction but not in the vertical plane. It should be noted that fulfillment of any of these relations is not required by the optimization process.

**Figure 5:**
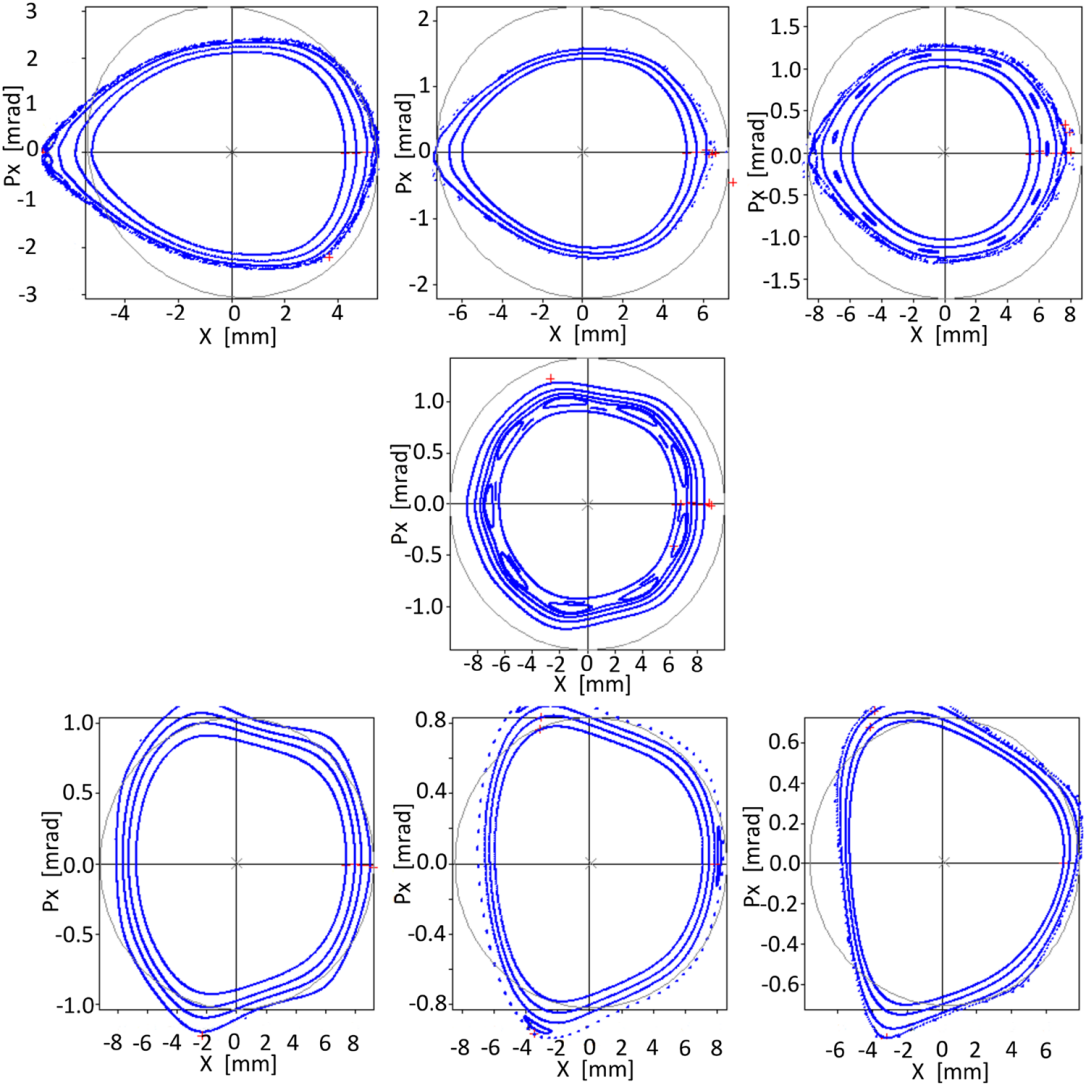
Phase spaces for various values of $\delta$. On the top row, for − 3, − 2, and −1 %. In the center for 0 % and in the lower row, for 1, 2, and 3 %. The optimization was only performed for the phase space corresponding to $\delta =0$ using the objective function $f_{obj1}^\delta$ of Eq. (21). The emittance of this operating point is 113 pm$\,\cdot \,$rad.

The first optimization using objective function $f^\delta _{obj1}$ considers only the phase space for $\delta =0$, as it was done in Ref.^40^, to assess the stability spread to values of $\delta \ne 0$, as in Figs. (12-17) of the mentioned reference. The spread of dynamical stability for $\delta \ne 0$, when the phase space at $\delta =0$ is optimized, is shown in Fig. 5.

For this first model, with an emittance of 113 pm$\,\cdot \,$ rad, the phase space at $\delta = 0$ has been optimized for several amplitudes, up to $x_0=7$  mm. However, stability is achieved a little beyond this amplitude where it is observed that invariant tori exist bounding resonances of high order.

It is interesting to note that, although the nonlinear surfaces related to the phase spaces for $\delta \ne 0$ did not intervene in this optimization process, the selected nonlinear parameter scheme also results in zones of stability in these spaces, for $x_0 \le 5.2$  mm in the range of $\delta \in (-\,3,3)\%$.

To compare with resonant techniques, Table 2 shows the output of the OPA Non-Linear Dynamics module with the set of sextupoles and octupoles that produces the orbits of Fig. 5, without further optimization. Several chromatic and geometric integrals appear with reduced values. Some integrated values of sextupole intensities are close to zero and these sextupoles could probably be discarded. Other values suggest sextupole coupling. The maximum intensity value of sextupoles is not high if we consider that the model has an emittance of 113 pm$\,\cdot \,$ rad. It is worth noting that the values of resonant integrals and sextupoles and octupoles integrated strengths are within acceptable ranges even though only horizontal space was considered in the optimization.

**Table 2:** Output of the OPA Non-Linear Dynamics module with the set of sextupoles and octupoles that produces the orbits of Fig. 5, without additional optimization.

Resonant term^17^	Value	Sextupole	$b_3L$
CrX lin	$-\,0.27 \;$	$SD2 \;$	$9.209 \;$
CrY lin	$-\,0.12 \;$	$FD2 \;$	$3.087 \;$
H21000	$0.96 \;$	$FD3 \;$	$0.001\;$
H30000	$3.42 \;$	$S1 \;$	$-\,5.341 \;$
H10110	$29.61 \;$	$S2 \;$	$-\,3.099 \;$
H10020	$34.23 \;$	$SE1 \;$	$-\,4.052 \;$
H10200	$11.22 \;$	$SE2 \;$	$3.225 \;$
H20001	$6.96 \;$	$S1S \;$	$0.371 \;$
H00201	$2.59 \;$	$S2S \;$	$11.511\;$
H10002	$0.03 \;$	$S1SH \;$	$0.098 \;$
CrX sqr	$-\,78.86 \;$	$SF2 \;$	$25.136 \;$
CrY sqr	$95.61 \;$	$S3 \;$	$-\,10.283 \;$
dQxx	$17.22.34 \;$	$S4 \;$	$-\,11.120 \;$
dQxy,yx	$5557.96 \;$	$FD22 \;$	$-\,7.650 \;$
dQyy	$-\,41627.03 \;$	$FD32 \;$	$0.034 \;$
H31000	$944.28 \;$	$SD32 \;$	$2.229 \;$
H40000	$542.69 \;$	$S1SH2 \;$	$0.053 \;$
H20110	$5201.34 \;$	$S2S2 \;$	$9.728 \;$
H11200	$1255.62 \;$	$S1S2 \;$	$-\,0.432 \;$
H20020	$3910.01 \;$	$SF1 \;$	$13.581 \;$
H20200	$882.98 \;$	$SD1 \;$	$-\,4.472 \;$
H00310	$1855.77 \;$	**Octupole**	$b_4L$
H00400	$180.66 \;$	$O2 \;$	$-\,166.82 \;$
CrX cub	$-\,2771.65 \;$	$O1 \;$	$-\,136.22 \;$
CrY cub	$-\,29475.51 \;$	$O3 \;$	$80.19 \;$
−	−	$O31 \;$	$491.70 \;$
−	−	$O11 \;$	$-\,293.85 \;$
−	−	$O21 \;$	$147.57 \;$

The model has an emittance of 113 pm rad.

### Using objective function $f_{obj1}^\delta$: optimizing three phase spaces for $\mathbf \delta =-\,2,\,0,\,2\,\%$

The idea behind the objective function of Eq. (21) is to promote similarity between the topologies of the phase spaces corresponding to nonlinear dynamics, with those of linear dynamics. In this section the range of influence that the functions $A^{(1)}_{ijkl}$ have on dynamic stability is explored. These functions represent the first contribution in $\delta$ to the polynomial quasi-invariant. The cell and point of operation are the same as in “[Sec Sec10]” section, which has an emittance of 113 pm$\,\cdot \,$rad.

**Figure 6:**
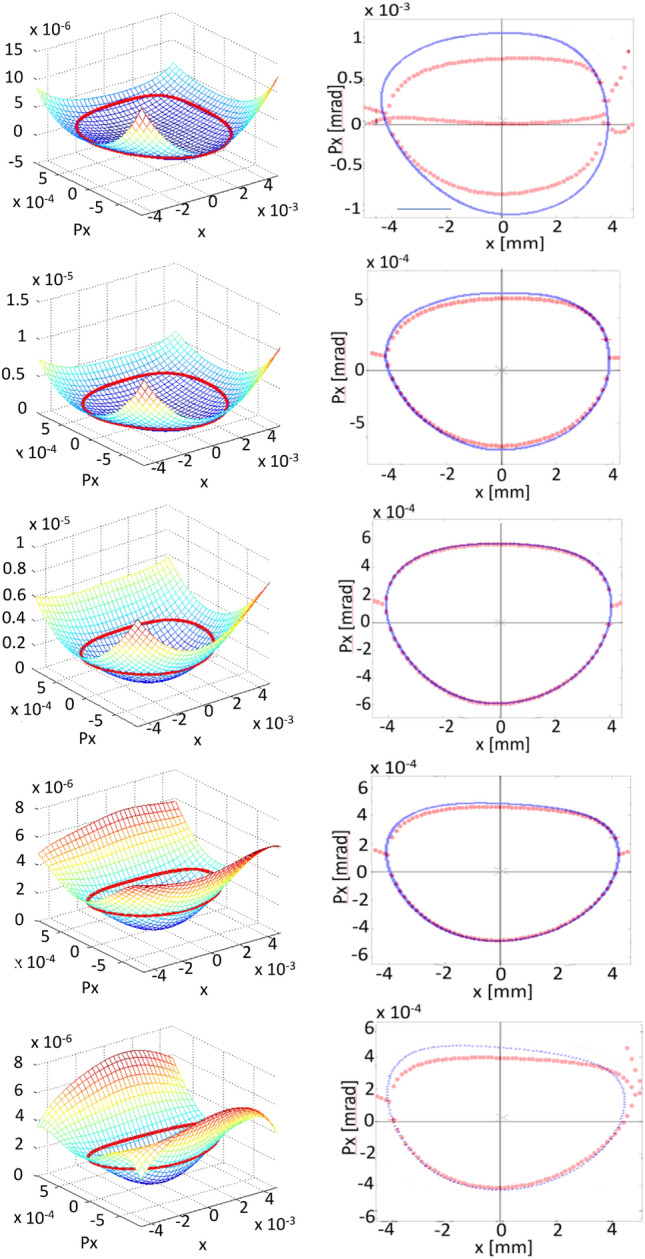
Phase spaces for various $\delta$ values. The first column of this figure shows the surfaces $S^\delta (x_{i},p_{xj})$ corresponding by rows to $\delta = -\,2,-\,1,0,1,$ and $2 \%$. The closed paths, confined by the basin, with amplitudes around 4 mm are shown in red. The second column shows the closed paths in phase space and their comparison with the tracking simulation with OPA, in blue. The optimization was performed including the phase spaces corresponding to $\delta =-\,2,0,$ and 2 %, using the objective function $f_{obj1}^\delta$ of Eq. (21).

In this case, the optimization process begins with the addition of two functions $f^\delta _{obj1}$, corresponding to off-momentum phase spaces $\delta =-\,2,\, 2\,\%$, to the objective function $f^{\delta =0}_{obj1}$. The amplitude used in the optimization is $x_0=7$ mm. Figure 6 shows the nonlinear surfaces, the trajectories in phase space, and their comparison with tracking simulation, through OPA, generated with the sextupole and octupole configuration obtained with the optimization process using genetic algorithms. A trajectory with amplitude close to 4 mm is shown in this figure. At $\delta =0$ (third row) very good overlap is observed between both curves. As $\delta$ moves away from 0 while less overlap is observed, the general form of the curve is similar, indicating that the theoretical contribution of $A^{(1)}_{ijkl}$ is less efficient in describing the orbits at large momenta, but it is a good measure of non-linearity. Further study is required to understand whether contributions from the functions $A^{(2)}_{ijkl}$, which are proportional to $\delta ^2$, are necessary to obtain better overlap at $\delta$ away from 0. Having a better theoretical representation of the nonlinear effects should improve the optimal selection of nonlinear elements that increase the dynamic aperture, including off-momentum particles.

**Figure 7:**
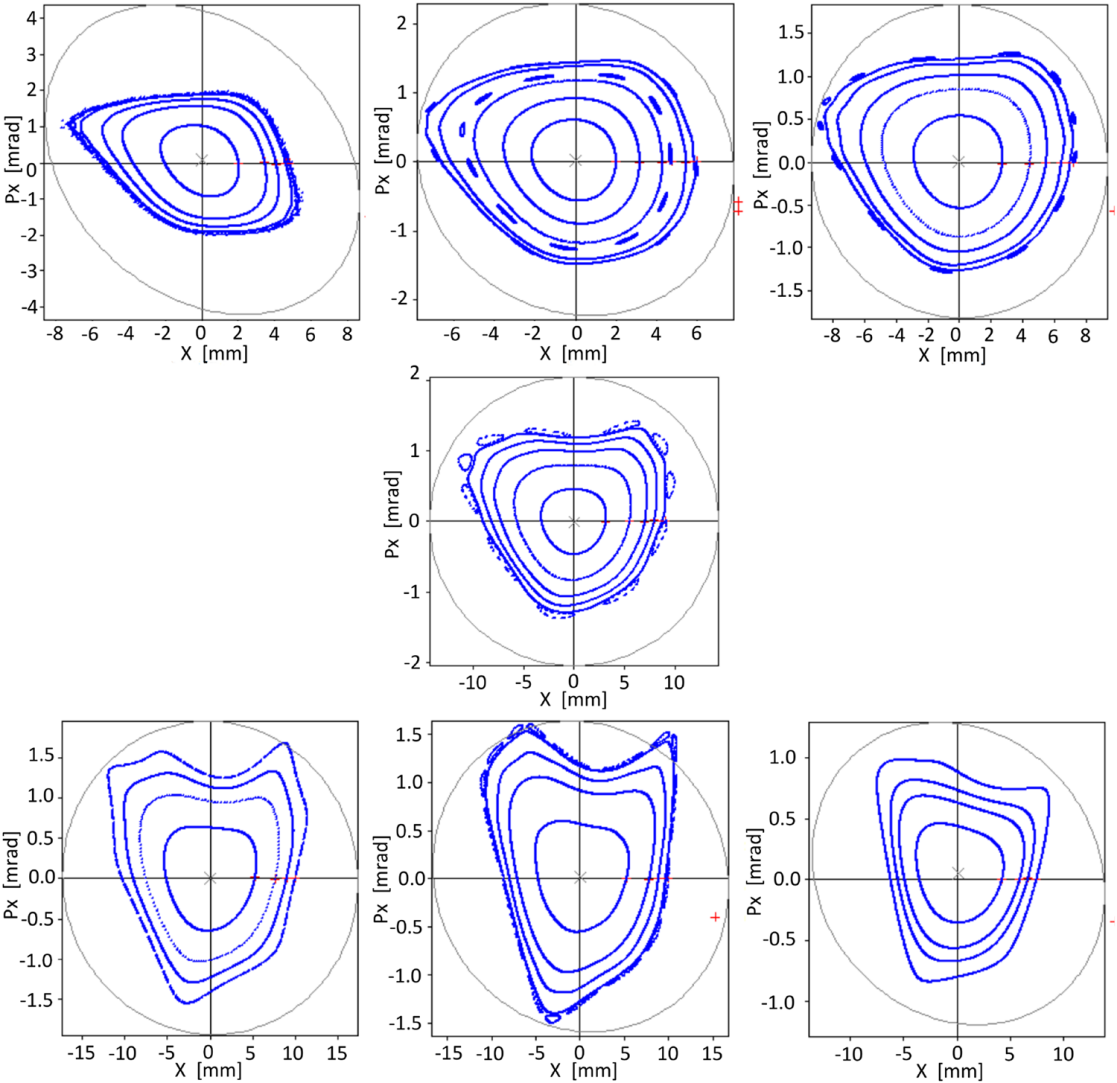
Phase spaces for various $\delta$ values. On the top row, for − 3, − 2, and − 1 %. In the center for 0 % and in the lower row, for 1, 2, and 3 %. The optimization was performed for the phase spaces corresponding to $\delta =-\,2,0,$ and 2 %, using the objective function $f_{obj1}^\delta$ of Eq. (21). The emittance of this operating point is 113 pm rad.

In Fig. 7 the most complete panorama is shown from the point of view of particle tracking. Using the set of sextupoles and octupoles found in the optimization process, OPA shows several closed orbits at different amplitudes and momenta. Although the optimization was carried out using only the phase spaces corresponding to $\delta =$ − 2, 0 and 2 % in the objective function, higher momenta have been explored with OPA also showing stability, for example, at $\delta =$ − 3, and 3 %. The optimization, done with an amplitude $x_0=7$ mm, provides stability up to 4.7 mm at − 3%, 5.9 mm at − 2%, 7.0 mm at − 1%, 8.7 mm at 0%, 10 mm at 1%, 9.7 mm at 2%, 7.5 mm at 3%. By optimizing the 3 phase spaces considered, it is found that there are phase spaces that have slight improvements in the stability zone, compared with one phase space optimization, in exchange for negatively affecting others.

These results show that by including the terms $A^{(1)}_{ijkl}(s)$ for $\delta \ne 0$, now implemented for treating off-momentum particles, there is stability up to 5.9 mm in the phase spaces for the range of $-\,2\%< \delta <2\%$ here optimized and, it is interesting to note that in the phase spaces of $-\,3\%< \delta <3\%$ there is stability up to 4.7 mm.

The results obtained may be due to multiple factors. Probably the comparison of linear and nonlinear surfaces in the objective function for phase spaces where $\delta \ne 0$ impose a significant restriction on the topology of the additional spaces, reducing their region of stability. Relaxing the restriction on the similarity of topologies between linear and nonlinear surfaces could lead to more promising results assuming that the $A^{(1)}_{ijkl}$ functions adequately approximate the nonlinear dynamics, behaviour that has been observed in particle tracking simulation for $\delta \in (\sim - 2, \sim 2)$. The introduction of contributions of $A^{(2)}_{ijkl}$ functions could also improve the description of the dynamics in the interval considered and probably extend the range of applicability to values of $\delta$ beyond this interval. Research in this direction is currently underway to increase the reliability of the $\delta$-dependent results.

### Using objective function $f_{obj2}^\delta$: optimizing the phase space for $\mathbf \delta = 0\%$

In this section the use of the objective function built with the Gaussian curvature, as shown in Eq. (27), will be studied. A major problem in synchrotron design is the choice of the appropriate operating point. In the course of this work, a new, lower-emittance operating point emerged, so it was decided to study it in more detail. For this purpose, some linear parameters were changed, including free space lengths. The parameters of this operating point are: the circumference is 484.452 m, the emittance is 89 pm rad, the chromaticities are $\xi _x=-\,85.91$ and $\xi _y=-\,56.17$; the tunes are $\nu _x=48.898$, $\nu _y=26.5386$. Again, we find that the phase advance in the *x* direction between dispersion bumps satisfy the $-\,I$ transformation since $\Delta \varphi _x=2.9972\, \pi$ but $\Delta \varphi _y=1.9922\, \pi$ is not a $-I$ transformation^54^.

**Figure 8:**
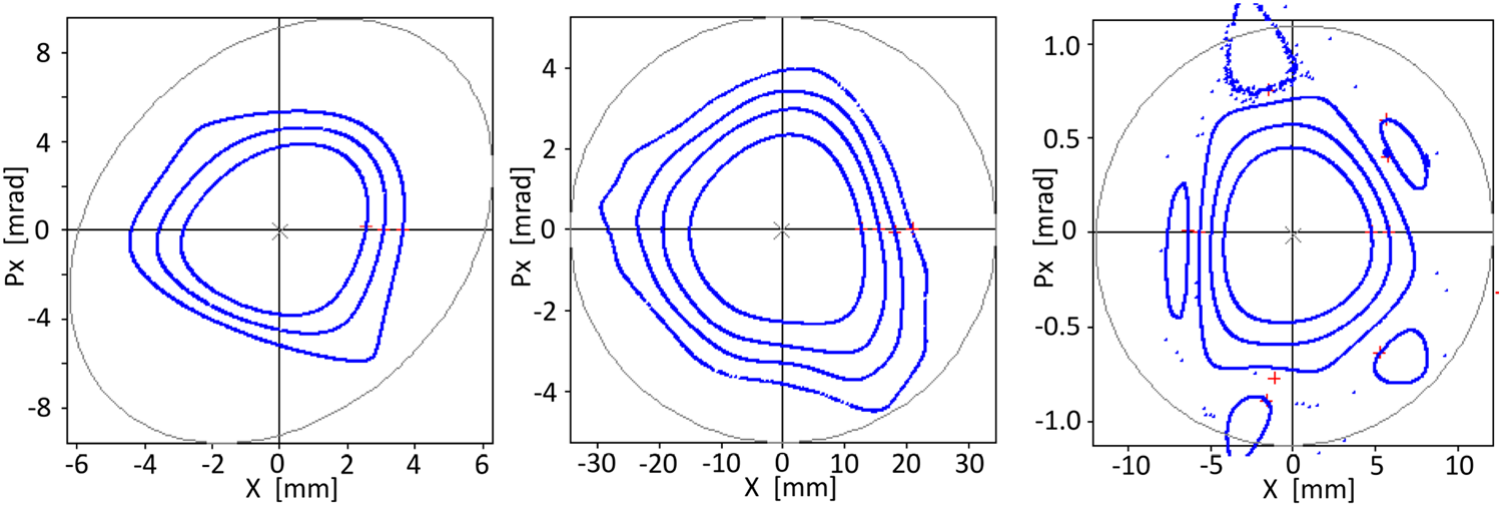
Phase spaces for three values of $\delta$ (- 3, 0, and 3 %). The optimization was performed only for the $\delta =0$ % phase space using the objective function $f_{obj2}^\delta$ of the Eq. (27). The phase spaces at $\delta = -\,3,\ 3 \%$ are included in this figure to show the stability spread for momenta close to $\delta =0$. Stability gradually decreases in phase spaces, between $\delta =0$ and $\mid \delta \mid \le 3$, (not shown).

Figure 8 shows the phase spaces obtained when the optimization is performed with the objective function of Eq. (27) that uses the Gauss curvature, at an oscillation amplitude of $$x_0=20 $$ mm, an amplitude that has been reached by gradually increasing the oscillation amplitude. It seems that this type-2 objective function converges faster when the curvature does not present very large values, which can be useful as a second approximation after the use of the objective function $$f_{obj1}^\delta $$. The tracking simulations in Fig. 8 show that the stability amplitude in the optimized phase space ($$\delta =0$$) can be significantly increased with this surface-based technique. Additionally, it is shown that, in an MBA design, which is more dynamically demanding than the model shown in ref.^40^, there is also an extension of stability, optimized at $$\delta =0$$, to values different from zero, as found in ref.^40^. The central figure in Fig. 8 shows a stable amplitude up to 20.8 mm, which is a promising amplitude for a synchrotron model of 89 pm$$\,\cdot \,$$rad. The stability zone is reduced for phase spaces that are away from $$\delta = 0$$; the obtained apertures are 3.65 mm for $$\delta =-\,3\%$$ and $$-\,5.6$$ mm for $$\delta =3\%$$, which could already be in useful ranges for the design.

### The importance of an appropriate operating point

**Figure 9:**
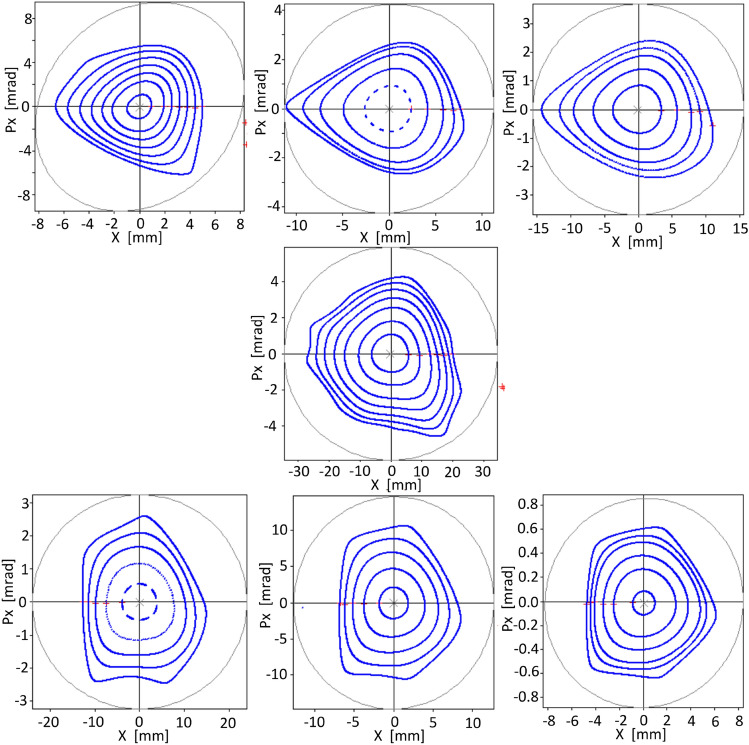
Phase spaces for − 3, − 2, − 1, 0, 1, 2, and 3 % $\delta$ values for the new operating point obtained by changing the quadrupoles as indicated in Eq. (39). Sextupoles and octupoles strengths correspond to those used in Fig. 8 without further optimization. The phase spaces for $\delta \ne 0$ show the stability spread for momenta close to $\delta =0$. Different scales are given to figures for clarity.

An asymmetry in the size of the stability zone for negative and positive values of $\delta$ has been frequently observed in the course of this study. An efficient procedure to control this asymmetry has to be devised. However, it has been observed in some models that if the tunes are modified, the mentioned asymmetry can be reduced. Two procedures have been used to select a new operating point. In the first case, new tunes were considered, and the corresponding quadrupole strengths were obtained with OPA TuneMatrix module. Several trials have been done selecting quadrupole strengths that produce lattices with relevant parameters within acceptable ranges. A second attempt has been done by allowing the cell external quadrupoles to take random strengths close to the initial design values, and then finding the best candidates in terms of emittance and chromaticities. This has been done using a Monte Carlo procedure that allows a quadrupole-strengths space sampling, computationally less expensive when compared with other protocols such as the GLASS method^55^. The results obtained by changing $\nu _x=48.898 \rightarrow 48.729$ and $\nu _y=26.5386 \rightarrow 26.7198$, adjusting the principal quadrupoles of the cell, is shown in Fig. 9. In this case, with the help of OPA, the new tunes are achieved by modifying the quadrupoles that are in the region of high dispersion in the form

39 $$\begin{aligned} \begin{aligned}KQ1 &= \,\,\,\,3.558418 \rightarrow \,\,\,\,3.551277 \\KQ2& = - 4.318293 \rightarrow - 4.323246 \\KQ3 &= -2.492566 \rightarrow - 2.495426 \\KQ4 &= \,\,\,\,2.757935 \rightarrow \,\,\,\,2.752401 \\KQ5 &= - 3.488500 \rightarrow - 3.492502 \\ \end{aligned} \end{aligned}$$

The new emittance and chromaticity parameters are respectively $\varepsilon _x=86$ pm$\,\cdot \,$rad and $\xi _x=-80.23$, $\xi _y=-56.72$. Figure 9 shows the effect of changing the operating point. The sextupoles have not changed with respect to Fig. 8, therefore, chromaticities have non zero values as previously requested. The stability zone of the phase space for $\delta =0$ does not suffer a great change as it remains at 20 mm, while at $\delta =-\,3\, \%$ it rises to 5 mm and at $\delta =3\, \%$ it is reduced to 4.7 mm, which slightly balances the stability for positive and negative values of $\delta$.

The sextupoles and octupoles intensities used in the calculation of the stability zones of Figs. 8 and 9 are given below. Taking the nonlinear multipoles from the full list (38), the corresponding magnitudes of $b_3$ and $b_4$ are given in the Eq. (40).

40 $$\begin{aligned} \begin{aligned} (&{\textbf {SE1}}, {\textbf {FD2}}, {\textbf {FD3}}, {\textbf {SD1}}, \underline{O2},{\textbf {S1}}, {\textbf {SF1}}, \underline{O1},\\&{\textbf {S2}}, \underline{O3}, {\textbf {SD1}}, {\textbf {SD3}}, {\textbf {S1S}}, {\textbf {S2S}}, {\textbf {S2S}}, \\&{\textbf {S1SH}}, {\textbf {S1SH}}, {\textbf {S2S}}, {\textbf {S2S2}}, {\textbf {S1SH2}}, {\textbf {S1SH2}}, \\&{\textbf {S2S2}}, {\textbf {S2S2}}, {\textbf {S1S2}}, {\textbf {SD32}}, {\textbf {SD2}}, \underline{O31}, {\textbf {S4}},\\&\underline{O11}, {\textbf {SF2}}, {\textbf {S3}}, \underline{O21}, {\textbf {SD2}}, {\textbf {FD32}}, {\textbf {FD22}}, {\textbf {SE2}} )\times length\\&=\\&(3.597, - 4.800, 0.081, - 11.807, 409.37, 0.805, 10.187, \\ {}&- 100.10, - 0.610, 169.94, - 11.807, 0.727, 0.251, 2.173, \\ {}&2.173, - 0.134, - 0.134, 2.173, 2.576, 0.031, 0.031, \\ {}&2.576, 2.576, 0.402, 1.106, - 6.952, 85.55, 2.611, - 84.31, \\ {}&4.844, 1.338, 66.78, - 6.952, - 0.291, - 2.458, 2.421). \end{aligned} \end{aligned}$$

At this level, we are interested in studying whether the previously obtained dynamic stability can be increased, using this new operating point, by optimizing only the phase space at $\delta =0$. For this, the two objective functions $f_{obj1}^\delta$ and $f_{obj2}^\delta$ will be used.

a)*Optimization using the *$f_{obj1}^\delta$ *for* $\delta =0$

**Figure 10:**
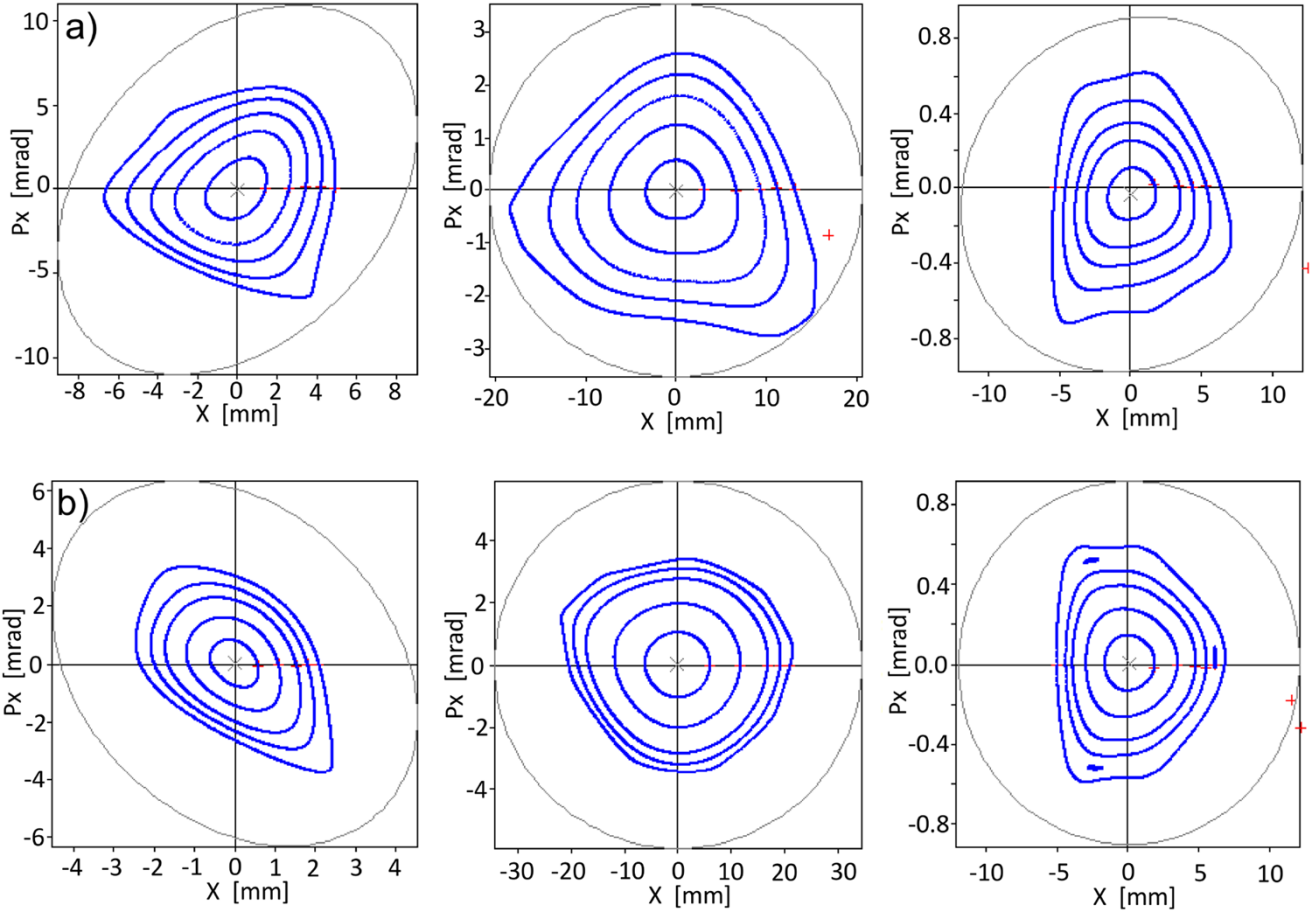
Phase spaces for − 3, 0, and 3 % $\delta$ values for the new operating point obtained by changing the quadrupoles as indicated in Eq. (39). Two different solutions were obtained optimizing sextupole and octupole strengths, contrary to what was shown in Fig. 9, with $f^\delta _{obj1}$ (**a**) and with $f^\delta _{obj2}$ (**b**).

The solution found during the optimization process of sextupoles and octupoles for this new operating point with 86 pm rad emittance using $f_{obj1}^\delta$ for $\delta =0$ is presented in Fig. 10a. Their maximum stability amplitudes are 4.9, 13.2, − 5.3 mm for $\delta =-3,0,3\%$ respectively. Again, as in Ref.^40^, when the phase space of $\delta =0$ is optimized, there is stability spillover into phase spaces of $\delta \ne 0$. An equivalent stability area is observed for off-momentum phase spaces. There are no significant changes in the stability amplitude for off-momentum phase spaces compared with Fig. 9.

b) *Optimization using Gauss curvature *$f_{obj2}^\delta$ *objective function for* $\delta =0$

Now the Gaussian curvature-based objective function $f^\delta _{obj2}$ will be applied to this new operation point.

The stability zone obtained by optimizing for $\delta =0$ are shown in Fig. 10b. The maximum oscillation amplitudes that define the stability regions are 2.1 mm, 21.1 mm and − 5 mm for $\delta =-\,3,0,3\%$ respectively. An improvement of the stability region for the on-momentum phase space and a reduction for off-momentum phase spaces can be appreciated when compared with the results obtained with $f^\delta _{obj1}$ (Fig. 10a). Both results show a good stability zone for on-momentum phase space and stability spread for the off-momentum phase spaces, for this MBA-based model. This spread has been also observed in Ref.^40^ for a third-generation synchrotron model. The off-momentum stability obtained for the MBA-model is rather limited. This probably indicates that the constraint used to achieve linear-like on-momentum dynamics may be too strong. A more relaxed condition may improve off-momentum stability. In the next section, a simple way to relax the condition imposed on the above objective functions is shown.

### Relaxing constraints

We consider that it is of interest to ask about the changes that can be produced in the stability of the phase spaces if the restrictions of the objective function are relaxed. In the previous case, the optimization process induces the Gaussian curvature of the nonlinear surface to be close to 4, which is the approximate value of this variable in the linear surface.

A slight decoupling can occur if, in the optimization, each principal curvature is separately required to comply with the relationship in Eq. (26), i.e. $\kappa _1= 2\gamma _x(0)$ and $\kappa _2=2\beta _x(0)$ in each phase space at fixed $\delta$. Both contributions are added to form the objective function

41 $$\begin{aligned} f^0_{obj3} &=  \sum _{j=1}^{n} \sum _{i=1}^{n} \left\{ \mid \kappa _1(x_i,p_{{x}{j}})-2\gamma _x(0)\mid \nonumber \right. \\ & \quad +\left. \mid \kappa _2(x_i,p_{{x}{j}})-2\beta _x(0)\mid \right\} , \end{aligned}$$

where the superscript 0 indicates that this function is for $\delta =0$ phase space.

**Figure 11:**
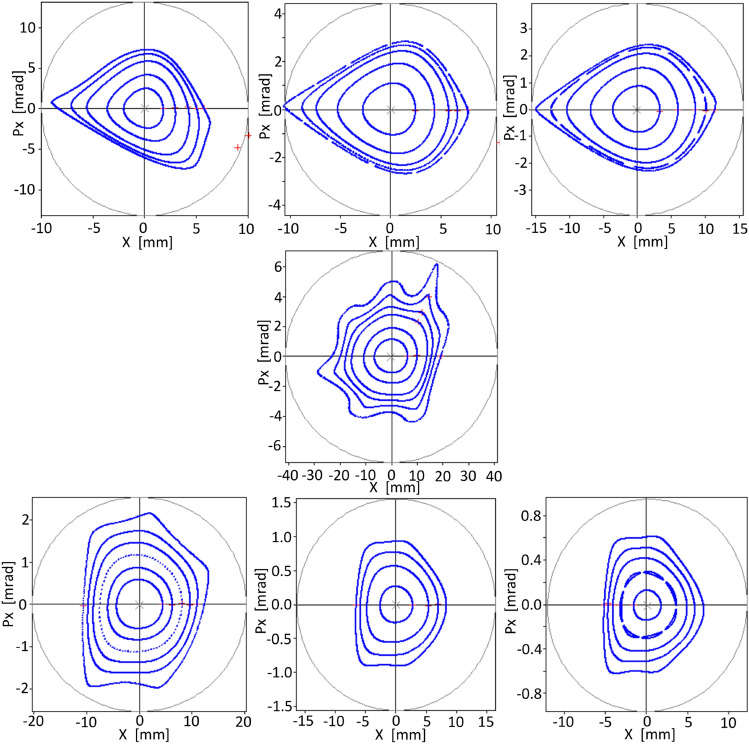
Phase spaces obtained by optimizing $f^0_{obj3}$ for $\delta =0$. They are shown in upper row for $\delta$ − 3, − 2, − 1 , in middle row for 0, and in bottom row for 1, 2, 3 %. When compared with Fig. 10b, the stability zone for negative and positive momentum deviations has now similar values, increasing the stability amplitude at $\delta <0$ momenta deviation.

**Figure 12:**
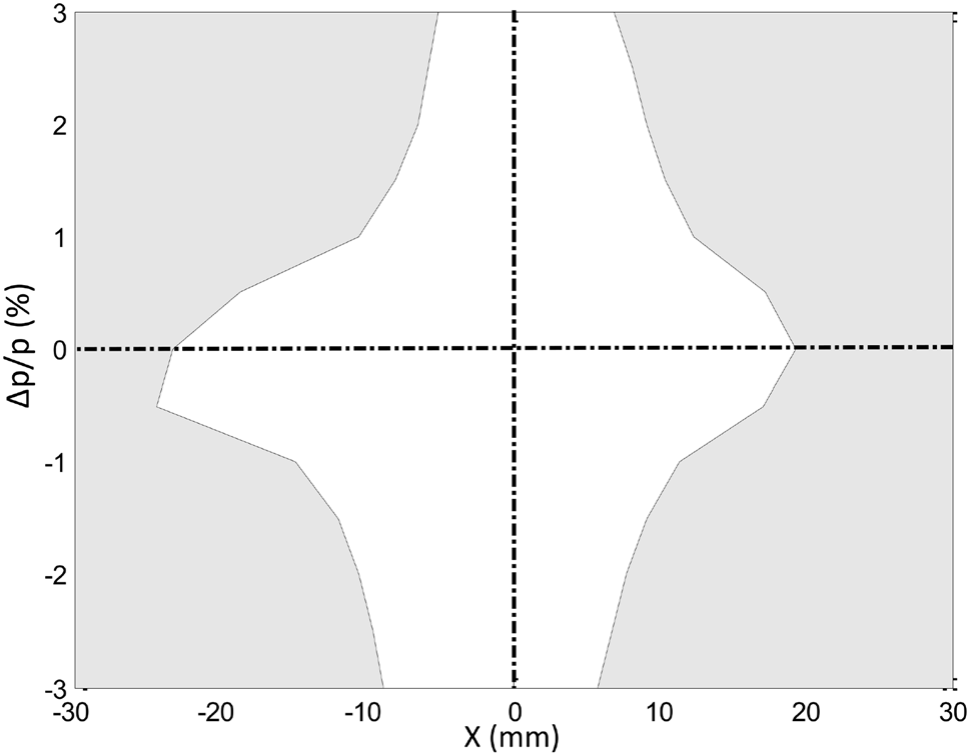
Space of momentum deviations $\delta$ vs horizontal oscillation amplitude x, showing the reduction of the oscillation amplitude as $\delta$ moves away from 0. The white area represents the stability zone of the solution shown in Fig. 11.

Starting from the solution shown in Fig. 10b, an optimization with this objective function was performed. The phase spaces found are shown in Fig. 11. The maximum amplitudes of the stability zone are 5.8 mm at − 3 %, 19.3 mm at 0 % and − 5.1 mm at 3 %. Good stability zone for each value of momentum $\delta$ is obtained and shown in Fig. 12. The zone of greatest stability is close to $\delta =0$ with amplitudes of horizontal stability $x\sim 20$ mm. When the value of $\delta$ moves away from 0, the stability zone decreases following a triangle-like geometry. Similar behaviour should be expected in other points around the cell; however, this analysis is beyond the scope of this work. With this scheme there is an increase in the stability zone for the $\delta \ne 0$ phase spaces compared with the solution shown in Fig. 10b. As a matter of fact, this objective function provides the best solution for the MBA-based model considered.

This result shows that reducing the constraints imposed on the objective function allows increasing the stability zone of the off-momentum phase spaces. Schemes of less restrictive objective functions are under study.

**Table 3:** Summary of the main parameters and values of the maximum stable amplitudes of the studied models, obtained with the objective functions $\textrm{f}_1=f^0_{obj1}, \textrm{f}_2=f^0_{obj2}, \textrm{f}_3={f}^0_{obj3}, \textrm{f}_\delta =\hspace{-.5cm}\underset{\delta \in \{- 2,0,2\%\}}{\sum }\hspace{-.5cm}f^\delta _{obj1}$, for three phase spaces ($\delta =-3,0,3\%$).

			Model	
	Circumference (m)	485.016	484.462	
	$\epsilon _x$ (pm rad)	113	89	86
	$\nu _x$	48.802	48.898	48.729
	$\nu _y$	27.653	26.5386	26.7198
	$\xi _x$	− 86.70	− 85.91	− 80.23
	$\xi _y$	− 64.01	− 56.17	− 56.72

$\delta$	Objective function	Maximum amplitud (mm)		
$-3\%$	$\textrm{f}_1$	5.2	–	4.9
	$\textrm{f}_2$	–	3.65	2.1
	$\textrm{f}_3$	–	–	5.8
	$\textrm{f}_\delta$	4.7	–	–
$0\%$	$\textrm{f}_1$	8	–	13.2
	$\textrm{f}_2$	–	20.8	21.1
	$\textrm{f}_3$	–	–	19.3
	$\textrm{f}_\delta$	8.7	–	–
$3\%$	$\textrm{f}_1$	− 5.6	–	-5.3
	$\textrm{f}_2$	–	− 5.6	− 5
	$\textrm{f}_3$	–	–	− 5.1
	$\textrm{f}_\delta$	7.5	–	–

Table 3 shows the maximum stability amplitudes of the models presented in the previous sections using the different objective functions in the optimization process.

**Figure 13:**
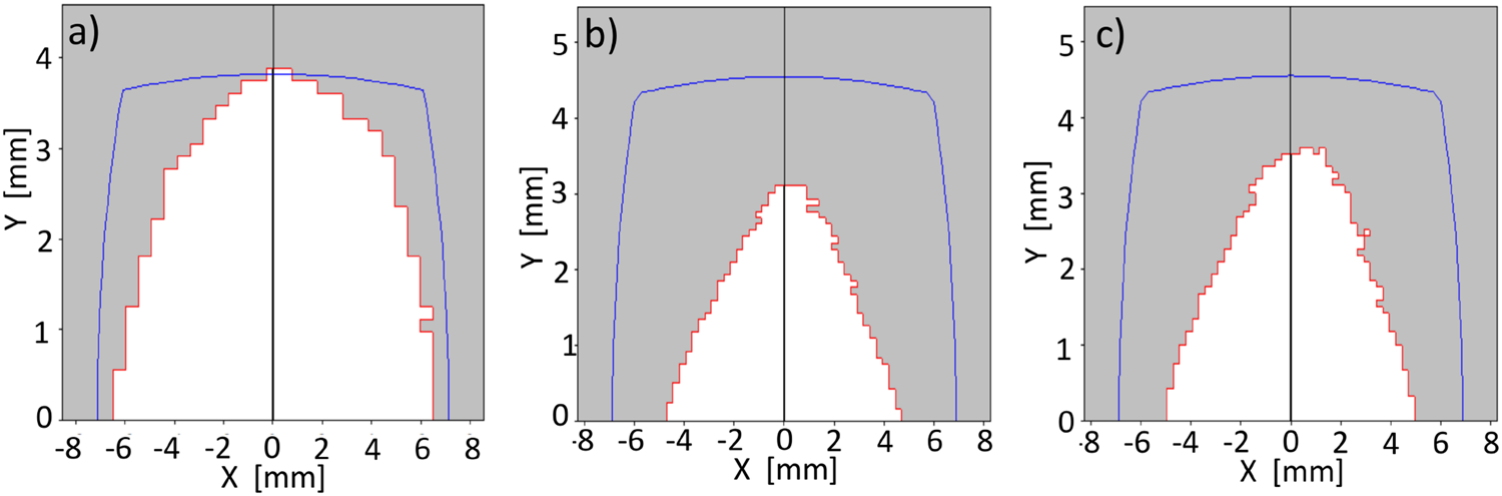
Dynamic apertures provided by OPA for $\delta =0$ for three cases of nonlinear magnetic multipoles optimization, (**a**) for the case of 113 pm rad of Fig. 5, (**b**) for the case of 86 pm rad of Fig. 10, (**c**) for the case of 86 pm rad of Fig. 11. The OPA code does not perform the dynamic aperture evaluation for $y<0.001$ mm, thus, data corresponding to $y=0$ are not depicted in these figures.

A more realistic analysis of the quality of the magnetic multipole sets found so far can be achieved with the OPA dynamic aperture calculation module, when using the multipole set optimized with the methods proposed in this work. Figure 13 presents dynamic apertures for some of the models, showing acceptable horizontal amplitudes for $$y\ne 0$$, despite the low emittances of the models and considering that the vertical dynamics is not optimized. It is worth noting the positive effect on the vertical space, as depicted in Fig. 13c, when the horizontal dynamic optimized set of multipoles employed in Fig. 12 is used. Figures 11 and 13 are calculated with the same set of multipoles optimized with the present theory. Figures 11 and 13 depict 1-D and 2-D results respectively. There is an apparent difference between both figures since the maximum amplitude $$x\sim \,19$$ mm of Fig. 11 for $$\delta =0$$ is not shown in Fig. 13c for $$y=0$$. The reason of this apparent discrepancy is that by default DA OPA module performs calculations for $$ y>0.001$$ mm and consequently the point $$(x,y) =(19.3, 0)$$ mm does not appear in Fig. 13c.

This is also evident in Table 2, where most of the resonant terms related to the vertical space reduce their values. The high values of the tune shift with amplitude *dQyy* and cubic chromaticity *CrY cub* terms could be due to the lack of vertical dynamics optimization. Nevertheless, the optimization performed with the method proposed in this paper generates a higher dynamic aperture than that obtained by using the OPA Nolinear Optimization module.

**Figure 14:**
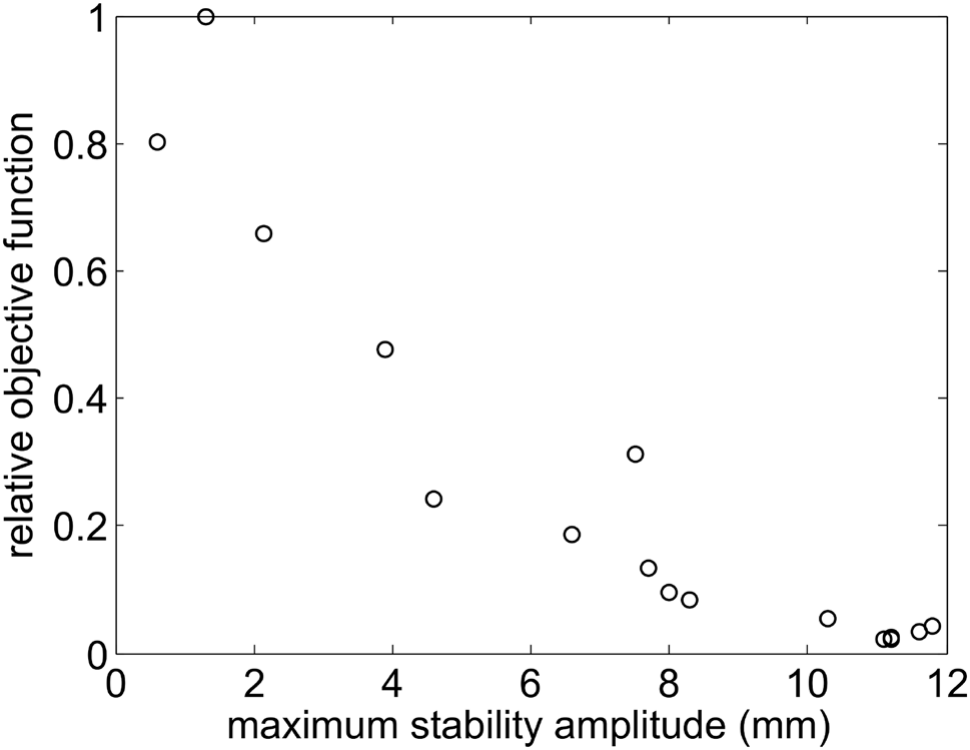
This figure shows, for a typical case, the general behavior between the objective function and the maximum stability amplitude. The vertical axis represents the objective function normalized to its initial value, and the horizontal axis represents the smallest |*x*| in mm, of the maximum stability amplitude, taken at $p_x=0$.

Additionally, the behavior of the maximum stability amplitude as the objective function reduces its value in the optimization process is presented. Figure 14 shows, for a particular optimization case, that the maximum stability amplitude increases as the normalized objective function takes smaller values. The horizontal axis is taken as the smaller of the maximum stability amplitude |*x*|. The relation between both variables is non-smooth, probably due to the sign ambiguity of *x* taken at $p_x=0$, and the fact that the objective function quantifies the difference between linear and non-linear surfaces. Although the dynamic aperture is not directly optimized, there is a clear relation between the value of the objective function and the maximum stability amplitude.

## Concluding remarks

A novel method based on nonlinear surfaces and surface curvatures, derived from quasi-invariant polynomials, has been successfully applied to optimize the nonlinear dynamics of a synchrotron MBA model. The primary objective was to identify maximum amplitude phase space trajectories. The complexity of the chosen MBA model, its low emittance and the high number of parameters to be optimized, make it suitable to assess method’s applicability. This evaluation suggest its robustness and suitability to optimize nonlinear dynamics in modern synchrotron light sources. The study has been extended to other lower emittance lattices not reported in this paper, showing equivalent results to those of this work, suggesting that an adequate description of the phase space is provided by the 8th degree polynomial quasi-invariant.

The control of nonlinear phenomena that appears in the studied synchrotron optimization, with a large number of free parameters (20 sextupoles and 6 octupoles), has been considered in order to test the tools used in the work, with encouraging results.

The optimization, based on the construction of a quasi-invariant polynomial of degree eight, considers that the nonlinear surfaces introduced in this work should be topologically similar to those of Courant Snyder or linear surfaces.

It has been demonstrated, at multiple operating points of the synchrotron model, that optimizing the on-momentum phase space leads to a wide stability region that extends to off-momentum phase spaces. The stability zone reduces as the momentum dispersion deviates from zero. Due to the broad on-momentum stability zone achieved, further off-momentum optimizations may benefit using the obtained nonlinear magnetic parameters as an starting point.

The nonlinear surfaces proportional to the momentum dispersion has been introduced, in order to adequately weigh off-momentum spaces. The results suggest that the description using $A^{(1)}_{ijkl}$ functions may be insufficient for $|\delta | \gtrapprox 2 \%$ and, probably, the use of $A^{(2)}_{ijkl}$ functions will be needed in order to properly describe this off-momentum range.

The improved results obtained with the introduction of the more relaxed function $f^0_{obj3}$ suggest that the constraint on the objective function from similarity between nonlinear and linear surfaces may be too restrictive and this condition could be relaxed. Work in this direction is ongoing.

### Supplementary Information

Supplementary Information.

## Data Availability

The data generated or analyzed during the current study are available from the corresponding author on reasonable request.
